# Intravenous methadone for perioperative acute and chronic pain management in Chinese adult cardiac surgical patients: A protocol for pilot randomized controlled trial

**DOI:** 10.1371/journal.pone.0323820

**Published:** 2025-06-02

**Authors:** Henry Man Kin Wong, Wai Tat Wong, Xiaodong Liu, Sylvia Siu Wah Au, Randolph Hung Leung Wong

**Affiliations:** 1 Department of Anesthesia and Intensive Care, The Chinese University of Hong Kong, New Territories, Hong Kong, China; 2 Division of Cardiothoracic Surgery, Department of Surgery, Prince of Wales Hospital, New Territories, Hong Kong, China; Public Library of Science, UNITED KINGDOM OF GREAT BRITAIN AND NORTHERN IRELAND

## Abstract

**Background:**

Postoperative pain is significant in cardiac surgical patients. Perioperative analgesia with intermittent administration of opioids can result in significant fluctuations in serum opioid concentrations. Methadone should provide a rapid onset and long-term pain relief upon a single intravenous dose at induction of anesthesia, and may reduce chronic postsurgical pain (CPSP) in cardiac surgical patients. The feasibility of using intravenous methadone in Chinese cardiac surgical patients, and its effect on acute and chronic pain management after cardiac surgery will be evaluated.

**Methods:**

A single-center, prospective, randomized-controlled pilot trial. Adult cardiac surgical patients will be randomized to receive 0.2 mg/kg methadone or morphine at induction of anesthesia. Patient-controlled analgesia morphine protocol, oral paracetamol and dihydrocodeine will be given for postoperative analgesia. Venous blood sampling for plasma methadone concentration will be obtained at regular intervals from study drug infusion to 96 hours after administration. The primary outcome will be a description of study feasibility, encompassing recruitment and retention, protocol adherence and stakeholder acceptability. Secondary outcomes include the time of ventilator weaning to spontaneous breathing, time of extubation, morphine requirements within 24 hours and 72 hours after surgery, time to first morphine rescue, postoperative pain scores, patient satisfaction, and length of stay in ICU and hospital. Opioid-related side effects including sedation, nausea and vomiting, and time to first bowel opening will be recorded. CPSP will be assessed with Neuropathic Pain Scale and Pain Catastrophizing Scale at 3 and 6 months after surgery.

**Discussion:**

Randomized controlled trials on intravenous methadone in cardiac surgical patients are scarce, with none in Chinese populations. This study, supported by plasma methadone concentration analysis, will establish a basis for future large-scale research aimed at improving recovery through optimized pain management.

**Clinical trial registration:**

ClinicalTrials.gov NCT05913284.

## Introduction

### Pain in cardiac surgery

Despite modern day improvements in pain treatment and availability of different analgesic modalities, suboptimal postoperative pain control remains an issue in cardiac surgical patients. Acute postoperative pain is common among cardiac surgical patients, particularly within the first 2 days after surgery, with reported at least moderate intensity [1]. There could be many facets for postoperative pain after adult cardiac surgery. Pain can be caused by surgical incisions and dissections, sternal fracture or incomplete bone healing, multiple drainage cannulas and chest tubes and sternal wound infections [2–4]. Poorly controlled acute postoperative pain is associated with adverse physiological outcomes that impair the recovery of cardiac surgical patients. It is associated with decreased patient satisfaction, delayed postoperative ambulation, and the development of chronic postsurgical pain (CPSP) [5]. The association between sternotomy pain and pulmonary complications has been observed, and the sympathetic activation secondary to pain can induce myocardial ischemia and arrhythmias [6–7]. Pain control has also been pointed out as one of the major concerns to cardiac surgical patients in intensive care unit [8]. Therefore, optimal acute pain control not only can improve clinical outcomes, but also improves patient satisfaction after cardiac surgery.

Postoperative pain that persists beyond the normal time for tissue healing is increasingly recognized as an important complication after various types of surgery. According to the International Association for Study of Pain, CPSP is defined as the persistence of pain at surgical site or referred area, at least 3 months following the surgical procedure [9]. CPSP is common after cardiac surgery. The reported incidence was 28% to 56% up to 2 years postoperatively [10–12]. Several mechanisms have been involved in the development of chronic pain after sternotomy. These include dissection, nerve entrapment by sternal wires, sternal retraction, ribs fractures, and intercostal neuralgia as a consequence of nerve damage during dissection of the internal mammary artery during coronary artery bypass graft (CABG) [2–3]. In addition, poorly controlled pain has been a general risk factor for the development of CPSP. All can stimulate the release of pro-inflammatory cytokines which sensitize the afferent nociceptive fibres to cause chronic pain. CPSP has the potential to impact daily functioning and quality of life of patients, as well as increasing the healthcare costs. CARDpain study reported that among those with CPSP, over 50% had significant pain-related interferences with activities of daily living (family and home responsibilities, recreation and employment) at 3, 6 and 12 months following cardiac surgery [13]. Therefore, apart from optimal acute pain control, it is equally important to prevent and manage CPSP, to ensure better satisfaction and quality of lives for our patients.

### Challenges in pain management

Intravenous opioids such as fentanyl and morphine have been the mainstay of perioperative analgesia for cardiac surgery, either by intermittent boluses or through a patient-controlled device. The primary problem with this mechanism of delivery is that significant fluctuations in serum opioid concentrations can occur, resulting in effects which range from inadequate analgesia to overdose and respiratory depression. These peaks and troughs of analgesia that occur with intermittent opioids administration may explain the suboptimal pain control during the initial postoperative period. In contrast to intermittent administration of short-acting opioids such as morphine and fentanyl, a single dose administration of methadone can be considered. Methadone was conventionally used in cancer and chronic pain management. It can be administered via oral, intravenous, and other parenteral routes. Despite being an often-used alternative to morphine, it remains relatively invisible in perioperative settings. Methadone is a unique opioid that may provide several important potential benefits for surgical patients in the perioperative period. It is a potent mu receptor agonist with a rapid onset and longest half-life (24–36 hours) of the clinically used opioids. According to a pharmacokinetic study [14], central nervous system effect site methadone concentration rapidly equilibrates with plasma concentrations, evidenced by a short lag time between plasma concentrations and effects (t_1/2_k_e0_ 4 minutes). This is comparable to the rapid onset and effect compartment equilibration of fentanyl and sulfentanil (5–6 minutes), and in contrast the slow onset time of morphine, where t_1/2_k_e0_ has been reported to exceed 4 hours [15]. In addition, as reviewed in an editorial, when methadone is administered at a dose of 20 mg or higher, the duration of analgesia approximates the half-life of 24–36 hours. Therefore, a single intravenous dose 20 mg administered to an adult at induction of anaesthesia should provide a rapid onset and significant pain relief up to 1–2 days postoperatively, which is the period reported to have the highest pain score after cardiac surgery. Methadone is also a N-methyl-D-aspartate (NMDA) receptor antagonist. It has been reported to possess anti-hyperanalgesic and anti-allodynic properties, that is important in preventing pain sensitization and the development of CPSP [16–17], which is of high risk in cardiac surgical patients.

### Side effects of methadone

Methadone shares the same opioid-related side effects as with many other opioids such as nausea and vomiting, drowsiness and respiratory depression. According to a meta-analysis in 2020 which included seven randomized controlled trials on intraoperative use of intravenous methadone [18], four studies reported no adverse events. One study reported that the patients who received intraoperative methadone experienced more sedation compared to control group at 24 hours after surgery. One study reported that the intraoperative morphine group had more sedation compared to methadone group during the postoperative period. Methadone was not shown to have a higher incidence of postoperative nausea and vomiting compared to the morphine group. Methadone has been reported to be associated with cardiac conduction abnormalities such as QT prolongation, QT interval dispersion and cardiotoxicity (Torsade de pointes) [19]. However, most of the cardiac-related side effects were seen in patients on prolonged or maintenance treatment with methadone. Significant dose-dependent QTc prolongation usually occurs at a high dose of methadone. A single injection of intravenous methadone at low dose is unlikely to result in significant cardiotoxicity. No cardiac disturbances were reported from the meta-analysis [18].

### Gaps in the literature

Only a few trials have studied intravenous methadone as an analgesic in surgical patients, in particular, there have not been many studies in the field of cardiac anaesthesia. A randomized controlled trial done by Murphy et al showed intraoperative methadone to be superior to fentanyl for patients undergoing cardiac surgery [20]. Methadone was demonstrated to reduce morphine consumption in the first 24 postoperative hours and improved pain scores at 12 hours after extubation compared to patients receiving fentanyl. There was 40% reduction in morphine requirement during the first 24 hours after extubation, and the severity of postoperative pain was decreased by 30–40% during the first three days after cardiac surgery. There was only one study comparing the analgesic efficacy between methadone and morphine given at the time of induction in cardiac surgical patients [21]. Methadone was shown to reduce opioid requirement at 24 hours postoperatively and significantly reduce the incidence of postoperative nausea and vomiting. So far there have not been any studies on using methadone for cardiac surgery in Chinese populations, and none on the role of methadone for prevention of chronic post-surgical pain in cardiac surgical patients. Substantial literature has demonstrated the ethnic differences in pain perception and endogenous pain modulation is postulated to be a mechanism for ethnic differences. Studies comparing Caucasians and Asians, such as Chinese and Indians, generally demonstrated lower pain tolerance in Asian populations [23–24].

The primary aim of this pilot trial is to evaluate the feasibility of the protocol and the effect of methadone on acute and chronic pain control after open cardiac surgery, compared with conventional opioid-based approach using morphine and fentanyl. In addition, the effects of methadone on opioids consumption, opioid-related side effects, patient satisfaction, postoperative extubation times, and length of stay in hospital and ICU will be determined. This trial will provide the foundation for a larger, adequately powered clinical trial, designed to evaluate the effect of methadone in pain management in cardiac surgery.

## Materials and methods

### Study population and design

The protocol of this pilot study was prepared using the SPIRIT checklist (S1 checklist in S1 Table). This is a single-centre, double-blinded, pilot randomized controlled trial conducted at Prince of Wales Hospital, a university teaching hospital with 1650 beds in Hong Kong. It has been registered on ClinicalTrial.gov under a registration ID NCT05913284, date of registration 8 August 2023. Ethics approval was obtained from the Joint Chinese University of Hong Kong-New Territories East Cluster Research Ethics Committee on 12 January 2023 (CREC Ref No. 2022.636-T). With an annual case load of 400 elective cardiac cases per year, the recruitment is anticipated to start on 01/05/2025 and to be completed by 30/09/2025. Data collection will be completed by 30/04/2026, and the results will be expected by June 2026. All elective cardiac surgical patients will be admitted to a 23-bed ICU for early postoperative care and monitoring with 1:1 nursing at all times, with an expectation of discharge from ICU to a high-dependency cardiac ward within 24 hours after surgery. Currently 350–400 adults undergo elective coronary artery bypass graft and/or valvular surgery each year.

### Randomization and concealment

Patients will be randomized to receive either 0.2 mg/kg methadone or morphine. The assigned medications will be added to a syringe containing saline, bringing the total volume to 50 mL. The randomization will be achieved by drawing sequentially numbered, coded sealed, opaque envelops, each containing the group assignment of either methadone or morphine. These sealed envelopes will be prepared by a third party who have no further involvement in the study. The study syringes containing the drug solution will be prepared by a nurse who is not involved in the study and will be labelled in a blinded manner. The primary care team, blinded to the group allocation, will perform all surgical procedures using standardized techniques. Anaesthesiologists and nurses, also blinded to group allocation, will record data intraoperatively and postoperatively in the ICU and at regular intervals in the cardiac wards.

### Eligibility criteria

We will include adult patients aged 18 or older who are undergoing elective coronary artery bypass graft (CABG), valve repair/replacement, or combined CABG/valve procedure via sternotomy, and who are expected to be extubated within 12 hours of surgery. Exclusion criteria include emergency surgery, aortic surgery, redo surgery, preoperative renal failure requiring renal replacement therapy or creatinine clearance less than 30 mL/min (calculated by the Cockcroft-Gault formula), liver dysfunction (liver enzymes exceeding twice the upper limit of normal), left ventricular ejection fraction less than 40%, requirement for mechanical hemodynamic support in the perioperative period, history of chronic pain or regular use of pain medications (except paracetamol and non-steroidal anti-inflammatory drugs), history of psychiatric illnesses or illicit drug use, intraoperative use of remifentanil and inability to provide informed consent.

### Anesthesia and interventions

All patients will receive standard monitoring for cardiac surgery. General anaesthesia will be induced with midazolam 0.01–0.05 mg/kg, fentanyl 2–5 mcg/kg and rocuronium 0.5–1 mg/kg to facilitate intubation with a single-lumen cuffed endotracheal tube. Anaesthesia will be maintained with sevoflurane and propofol infusion, targeting a Bispectral Index of 40–60. The study drug (either 0.2 mg/kg methadone or morphine) in blind labelling will be administered at the time of induction via intravenous infusion over 30 minutes. The dosages of the study drugs are chosen and are considered appropriate based on previous studies investigating the management of sternotomy pain after cardiac surgery [20–22]. No further morphine will be administered throughout the operation. No other analgesics (paracetamol, non-steroidal anti-inflammatory agents, dexmedetomidine, ketamine), steroids, or antiemetics will be given intraoperatively. Hypertension will be managed by increasing the rate of propofol infusion or concentration of sevoflurane, or by nitroglycerin boluses if the Bispectral Index drops below target. The postoperative analgesia protocol is identical in both study groups. Patient-controlled analgesia (PCA) morphine will be prescribed for 72 hours after operation for postoperative analgesia. Oral analgesics, including paracetamol one gram every six hours and dihydrocodeine 30 mg three times daily, will be prescribed by the parent surgical team. Antiemetic (intravenous ondansetron 4 mg every eight hours) will be prescribed on an as-needed basis, and rescue analgesics, in addition to the protocol regimen, may be prescribed as needed by the parent surgical team and pain team. At the end of the operation, patients will be kept sedated with propofol infusion to ICU. Propofol infusion will be discontinued upon admission to ICU to facilitate weaning from the ventilator. Adaptive Support Ventilation (ASV) is used in ICU for weaning, adjusting ventilation parameters based on the patient’s lung mechanics and effort. Pain will be assessed by nurses in the ICU four hours after stopping sedation, then once every two hours. Upon extubation, patient pain scores and sedation levels will be assessed at 15 minutes, 8, 12, 24, 48, and 72 hours post-extubation. One milligram of morphine will be administered via PCA to patients if pain of more than mild severity is noted. Any nausea or vomiting, and use of rescue antiemetics, will be documented.

### Outcome measures

The primary outcome will be a description of study feasibility, encompassing recruitment and retention, protocol adherence and stakeholder acceptability. A document analysis will be performed to determine the number of patients approached for the study, the number who meets the recruitment criteria, and the number who provides informed consent, receives the intervention and completes postoperative follow-up. A qualitative approach will be used to determine the protocol adherence, stakeholder acceptability of the protocol, and the barriers and enablers to the study. Clinician unfamiliarity and public stigmatization can result in resistance to the use of methadone for perioperative pain control. In addition, due to the long duration of action of methadone and the absence of clinical data in the Chinese cardiac surgical population, the effects of prolonged sedation and respiratory suppression, which may affect ventilation weaning and extubation, must be observed. The acceptability and perceived trial feasibility will depend on qualitative feedback from anaesthesiologists and cardiac surgeons.

A larger trial is deemed feasible if the following progression criteria are met: (1) successful randomization of enrolled patients to the methadone arm (intervention group) and the morphine arm (control group); (2) successful enrolment of 60 participants within 12 months; (3) at least 80% of the recruited participants with the postoperative outcomes captured at 24 hours after extubation; (4) at least 80% of the recruited participants with complete capture of postoperative outcomes up to 72 hours after extubation, and (5) sufficient retention proportion (≥ 85% of participants with data captured within 72 hours after extubation) at 3 and 6 months after surgery for assessment of chronic postsurgical pain; (6) any risks or safety issues arising from the use of methadone in cardiac surgical patients, such as prolonged QTc and cardiac arrhythmias not explained by surgical procedures or patient comorbidities; (7) any concern raised by the anaesthesiologists and cardiac surgeons in their qualitative feedback. All these feasibility and acceptability measures will inform the decision to proceed with the definitive trial in the future.

The secondary outcomes are the time to successful weaning to spontaneous breathing using ASV; morphine consumption within 24 and 72 hours after extubation, pain score at rest and during coughing, and patient satisfaction with pain management within 72h after the extubation; time to first morphine rescue; opioid-related side effects including the number of episodes of postoperative nausea and vomiting; time to first bowel movement; the recovery from surgery measured by length of ICU and hospital stay; and psychological distress related to pain at 3 and 6 months after surgery. Psychological distress will be assessed using the Neuropathic Pain Scale and the Pain Catastrophizing Scale.

### Data collection

**Fig 1:**
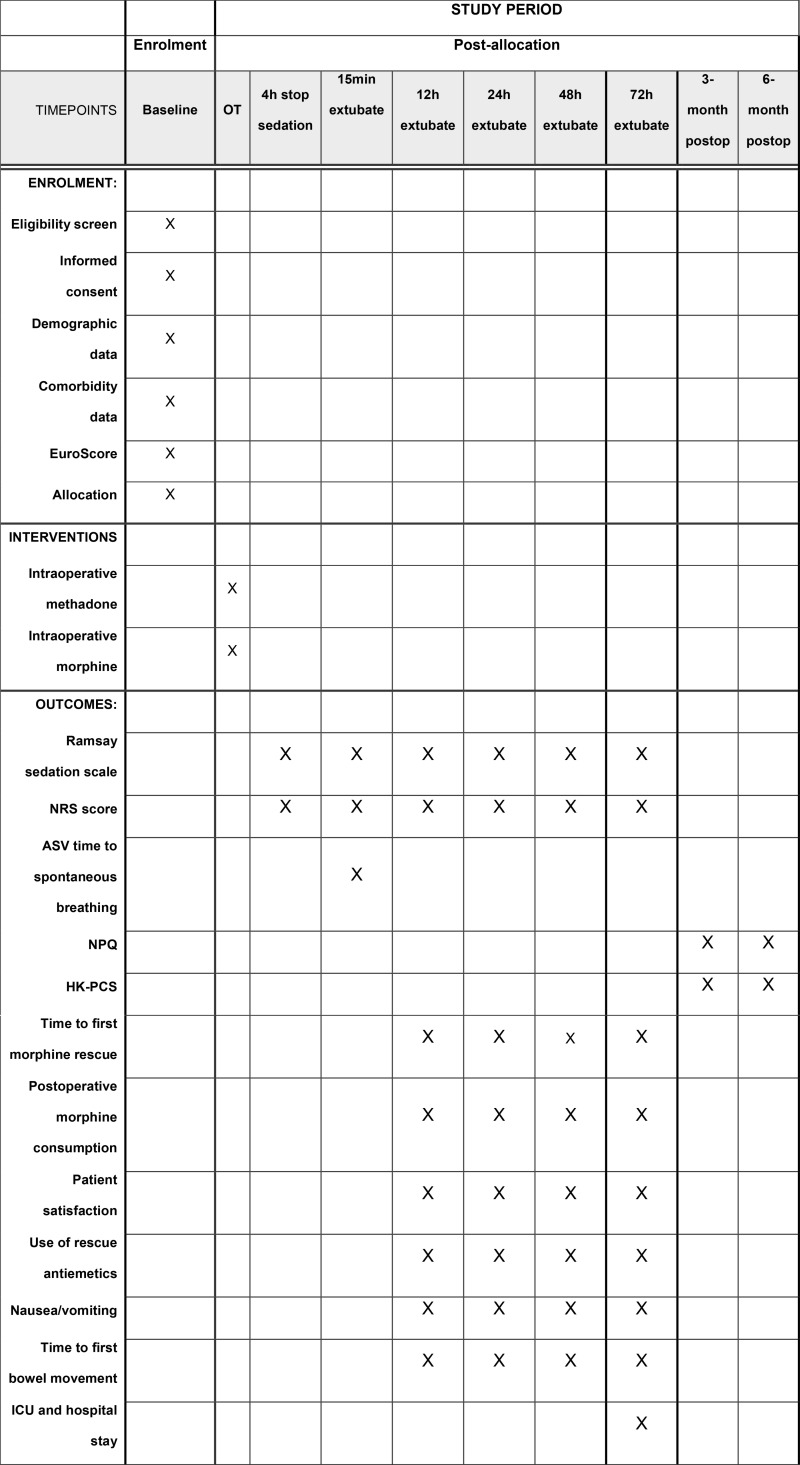
Timeline of assessments. NRS, numerical rating scale; ASV, adaptive support ventilation; NPQ, Neuropathic Pain Questionnaire; HK-PCS, Pain Catastrophizing Scale Hong Kong version.

The timeline of assessments are shown in Figure 1 (Fig 1). Patients will be screened for eligibility on the day prior to surgery. Eligible patients will receive patient information sheets outlining the main aspects of the trial. Research nurses will then discuss with the patients, elaborating on the information provided in the sheets. Written informed consent will be obtained from patients who agree to participate. All data will be collected by research team members blinded to group assignment. Patient demographics and body mass index will be recorded. The time to successful weaning to spontaneous breathing using ASV will be recorded. Pain scores at rest and during coughing will be quantified using the Numerical Rating Scale (NRS) from 0 to 10, where zero represents no pain and 10 represents the worst imaginable pain. These assessments will be performed at 15 minutes after tracheal extubation and at 8, 12, 24, 48 and 72 hours post-extubation. The time to first morphine rescue (in minutes after surgery) will be measured. The level of sedation will be measured using the Ramsay Sedation Scale (1 = anxious, agitated or restless, or both; 2 = co-operative, oriented and tranquil; 3 = respond to command only; 4 = exhibit brisk response to light glabellar tap or loud auditory stimulus; 5 = exhibit sluggish response to light glabellar tap or loud auditory stimulus; 6 = exhibits no response). Episodes of nausea and vomiting, and whether antiemetics are prescribed will also be recorded at these time points. Concurrently, patients will rate their overall satisfaction with pain management on a verbal analogue scale (0 = worst possible, 100 = best possible). The Chinese version of the Neuropathic Pain Questionnaire (NPQ) [25] will be used to evaluate the presence of neuropathic pain and the extent to which pain interferes with physical, emotional, and sleep functioning at 3 and 6 months after surgery. Pain Catastrophizing Scale (HK-PCS) [26] will be used to assess patients’ negative cognitive-affective response to pain at 3 and 6 months after surgery.

The following medical and surgical data during the hospital stays will be extracted from patient charts:

1. Patient demographics (age, gender, EuroScore)
2. Type of surgery
3. Duration of surgery and duration of cardiopulmonary bypass
4. ASV time to spontaneous breathing
5. Total morphine consumption at 24 and 72 hours after surgery
6. Episodes of nausea and vomiting, and use of rescue antiemetics
7. Length of ICU and hospital stay

### Blood sampling for methadone level

The blood sample will be taken by a research nurse or clinicians not involved in the study. The list anaesthesiologist and the parent surgical team, blinded to group assignment, will be informed of the blood sampling from the study participants without knowing the indication of the blood sampling. Venous blood samples (5 mL) will be obtained at 0, 30 and 60 minutes after drug administration, and at 1.5, 2, 3, 4, 5, 6, 7, 8, 10, 12, 24, 48, 72 and 96 hours after dosing. Plasma concentration of methadone will be determined by LC-MS/MS (ESI pos). The internal standard (7-dimethylamino-5,5-diphenyl-4-octanone, 2.5ng) will be added to plasma (0.5 mL), which will be acidified and then processed by SPE (Waters Oasis MCX cartridges).

### Sample size calculation

Based on the recommendations from Whitehead et al [27], a sample size of 60 patients (30 per arm) would provide an adequate estimate of the treatment effect to minimize the sample needed for a future RCT, assuming 90% power, a small-medium effect size of 0.3, and to account for 10% lost to follow-up.

There are approximately 400 cardiac surgical patients per year at the study hospital. The authors anticipate that half of the patients will fit our recruitment criteria. Given that intravenous methadone is a novel drug for acute pain management in Hong Kong, it is estimated that half of the patients will agree to participate. With an expected drop-out rate of less than 25%, it should be able to achieve the target sample size of 60 patients. With N = 60 participants recruited, retention of 80% can be estimated to within approximately 11% with 95% confidence. To estimate the sample size for a full study, the mean (standard deviation) morphine consumption for postoperative morphine requested at 24 hours after extubation, one of the secondary outcomes, for both study groups will be calculated based on intention-to-treat and per-protocol analysis. Intention-to-treat is defined as study patients who randomized to receive the study drug. All the secondary outcomes including the morphine consumption within 24 hours will be analyzed and reported on intention-to-treat basis. Informed by this value, the anticipated effect size and sample size will be calculated using G*Power 3.1, with 90% power, alpha 0.05 (two-tailed test), and a 20% attrition rate.

### Data analysis

Feasibility outcomes will be reported in percentages and/or counts. Barriers to using methadone in cardiac surgery will be described. Complication rates and adverse events will be reported as percentages and/or counts. Categorical data are reported as numbers and percentages. Continuous variables are reported as mean (standard deviation) or median (interquartile range). Secondary outcomes by treatment groups will be presented with 95% confidence intervals. SPSS 27.0 software (IBM Corp, Armonk, NY) will be used for data analysis. Pilot feasibility data will be used to inform a fully powered randomized controlled trial.

### Ethics, data management and dissemination

Patients will be screened for recruitment on the day prior to surgery, and the risks and benefits of the study will be explained. Written informed consent will be obtained from the patient. Patients may withdraw from the project at any time without prejudice. Study participants will be assigned a unique code as identifier throughout the study. All data will be entered into an electronic system by research team members who are trained in data entry. To ensure accurate data entry, a second member of the research team will check data entry. Data collection and study conduct will be monitored with the research team on weekly basis to ensure protocols are implemented consistently. All data will be kept confidential and maintained on a password-protected computer and in locked filing cabinets within the secure offices of the Department of Anesthesia and Intensive Care. Digital files will be securely deleted, and paper documents shredded, after 15 years. Only group data will be published. Access to data will be restricted to study investigators. Approval for the project has been obtained from The Joint Chinese University of Hong Kong-New Territories East Cluster Clinical Research Ethics Committee. The study will adhere to local laws, Declaration of Helsinki, International Council for Harmonization of Technical Requirements for Pharmaceuticals for Human Use Good Clinical Practice and Institutional Policies. All adverse events associated with the study drug will be recorded by the research team and reported to the trial management committee. The trial management committee, comprising external and independent clinicians, will review all events within 48 hours and discuss them at regular trial committee meetings. The results of this study will be disseminated at international conferences and in peer-reviewed journals.

## Discussion

Acute pain and chronic postsurgical pain are common in cardiac surgery. A single dose of methadone at induction has been shown to decrease acute postoperative pain and reduce perioperative opioid consumption when compared with standard of care using morphine and fentanyl [20–21]. This is likely the result of its long half-life and the NMDA receptor antagonist effect. By reducing perioperative opioids and antagonizing NMDA receptors, methadone also has the potential to reduce chronic postsurgical pain after cardiac surgery. However, the quality of evidence on intraoperative methadone use during cardiac-related procedures is limited due to the small sample size and the lack of diversity in demographics of patients. Three double-blinded randomized controlled trials were included in a systematic review on intraoperative methadone use in cardiac surgery [28], and one of the studies was a retrospective review that carried a risk of bias. Due to the limited number of studies reported, and none in Chinese population, more data is needed to evaluate the use of methadone in cardiac surgery.

The optimal dose of intraoperative methadone for cardiac surgical patients remains undetermined. Higher doses (≥ 20 mg) can provide analgesia for up to 24–36 hours [29]. A systematic review suggested that 0.1 to 0.3 mg/kg intraoperative methadone decreased both opioid use within the first 24 hours after surgery and pain scores without increasing opioid-related adverse events, which was compatible with our findings [28]. However, heterogenicity in study design and small sample sizes hinder definitive conclusions. Murphy et al demonstrated that intraoperative methadone at 0.3 mg/kg resulted in lower pain scores and less postoperative opioid use compared to fentanyl in CABG patients without increased side effects [20]. A recent retrospective dose-response study found that higher methadone doses (>0.3mg/kg) were associated with increased opioid-related side effects including PONV, delirium, and longer hospital stay. Notably, both low-dose (0.25 mg/kg) and medium-dose (0.25 to 0.3 mg/kg) methadone groups demonstrated reductions in pain scores up to postoperative day 7 and opioid requirements up to postoperative day 4, suggesting that lower doses might be adequate for postoperative analgesia in cardiac surgical patients. Given the potential for opioid-related adverse events, an ideal methadone dose should balance analgesic efficacy with the risks and side effects of opioid use, aligning with published guidelines on pain management and opioid stewardship in cardiac surgery [30]. Carvalho et al randomized cardiac surgical patients to receive either 0.1 mg/kg methadone or morphine at the end of surgery [31]. Despite lower pain scores in the methadone group at 24 hours after surgery, there were no difference in pain scores at 12 and 36 hours after surgery. Patients receiving methadone had a significantly shorter time to first analgesic request. A retrospective cohort study in CABG patients receiving 0.2 mg/kg methadone as part of multimodal analgesia found lower pain scores and postoperative opioid use until postoperative day 2 [32].

This pilot trial evaluates the feasibility of using single administration of methadone at 0.2 mg/kg at anesthesia induction surgical patients. The results of this trial will provide the data to support future large-scale randomized controlled trials regarding the analgesic efficacy of a lower-dose intraoperative methadone in cardiac surgery, aligning with opioid stewardship recommendation in cardiac anesthesia.

## Supporting information

S1 Table SPIRIT checklist. (DOCX)

S1 File Project submitted to the ethics committee. (DOCX)

S2 File Opinions from the ethics committee. (PDF)

## References

[1] Mueller XM, Tinguely F, Tevaearai HT, Revelly JP, Chioléro R, von Segesser LK. Pain location, distribution, and intensity after cardiac surgery. Chest. 2000;118(2):391–6. doi: 10.1378/chest.118.2.391 10936130

[2] Sharma AD, Parmley CL, Sreeram G, Grocott HP. Peripheral nerve injuries during cardiac surgery: risk factors, diagnosis, prognosis, and prevention. Anesth Analg. 2000;91(6):1358–69. doi: 10.1097/00000539-200012000-00010 11093980

[3] Defalque RJ, Bromley JJ. Poststernotomy neuralgia: a new pain syndrome. Anesth Analg. 1989;69(1):81–2. 2765029

[4] Kleiman AM, Sanders DT, Nemergut EC, Huffmyer JL. Chronic poststernotomy pain: incidence, risk factors, treatment, prevention, and the anesthesiologist’s role. Reg Anesth Pain Med. 2017;42(6):698–708. doi: 10.1097/AAP.0000000000000663 28937533

[5] Kehlet H, Jensen TS, Woolf CJ. Persistent postsurgical pain: risk factors and prevention. Lancet. 2006;367(9522):1618–25. doi: 10.1016/S0140-6736(06)68700-X 16698416

[6] Puntillo K, Weiss SJ. Pain: its mediators and associated morbidity in critically ill cardiovascular surgical patients. Nurs Res. 1994;43(1):31–6. 8295837

[7] Roediger L, Larbuisson R, Lamy M. New approaches and old controversies to postoperative pain control following cardiac surgery. Eur J Anaesthesiol. 2006;23(7):539–50. doi: 10.1017/S0265021506000548 16677435

[8] Chyun D. Patients’ perceptions of stressors in intensive care and coronary care units. Focus Crit Care. 1989;16(3):206–11. 2737343

[9] Werner MU, Kongsgaard UE. I. Defining persistent post-surgical pain: is an update required? Br J Anaesth. 2014;113(1):1–4. doi: 10.1093/bja/aeu012 24554546

[10] Eisenberg E, Pultorak Y, Pud D, Bar-El Y. Prevalence and characteristics of post coronary artery bypass graft surgery pain (PCP). Pain. 2001;92(1–2):11–7. doi: 10.1016/s0304-3959(00)00466-8 11323122

[11] Kalso E, Mennander S, Tasmuth T, Nilsson E. Chronic post-sternotomy pain. Acta Anaesthesiol Scand. 2001;45(8):935–9. doi: 10.1034/j.1399-6576.2001.450803.x 11576042

[12] Meyerson J, Thelin S, Gordh T, Karlsten R. The incidence of chronic post-sternotomy pain after cardiac surgery--a prospective study. Acta Anaesthesiol Scand. 2001;45(8):940–4. doi: 10.1034/j.1399-6576.2001.450804.x 11576043

[13] Choinière M, Watt-Watson J, Victor JC, Baskett RJF, Bussières JS, Carrier M, et al. Prevalence of and risk factors for persistent postoperative nonanginal pain after cardiac surgery: a 2-year prospective multicentre study. CMAJ. 2014;186(7):E213-23. doi: 10.1503/cmaj.131012 24566643 PMC3986330

[14] Inturrisi CE, Colburn WA, Kaiko RF, Houde RW, Foley KM. Pharmacokinetics and pharmacodynamics of methadone in patients with chronic pain. Clin Pharmacol Ther. 1987;41(4):392–401. doi: 10.1038/clpt.1987.47 3829576

[15] Dershwitz M, Walsh JL, Morishige RJ, Connors PM, Rubsamen RM, Shafer SL, et al. Pharmacokinetics and pharmacodynamics of inhaled versus intravenous morphine in healthy volunteers. Anesthesiology. 2000;93(3):619–28. doi: 10.1097/00000542-200009000-00009 10969293

[16] Gagnon B, Almahrezi A, Schreier G. Methadone in the treatment of neuropathic pain. Pain Res Manag. 2003;8(3):149–54. doi: 10.1155/2003/236718 14657982

[17] Santiago-Palma J, Khojainova N, Kornick C, Fischberg DJ, Primavera LH, Payne R, et al. Intravenous methadone in the management of chronic cancer pain. Cancer. 2001;92(7):1919–25. doi: 10.1002/1097-0142(20011001)92:7<1919::aid-cncr1710>3.0.co;2-g 11745266

[18] Kendall MC, Alves LJ, Pence K, Mukhdomi T, Croxford D, De Oliveira GS. The effect of intraoperative methadone compared to morphine on postsurgical pain: a meta-analysis of randomized controlled trials. Anesthesiol Res Pract. 2020;2020:6974321. doi: 10.1155/2020/6974321 32280341 PMC7140144

[19] Alinejad S, Kazemi T, Zamani N, Hoffman RS, Mehrpour O. A systematic review of the cardiotoxicity of methadone. EXCLI J. 2015;14:577–600. doi: 10.17179/excli2015-553 26869865 PMC4747000

[20] Murphy GS, Szokol JW, Avram MJ, Greenberg SB, Marymont JH, Shear T, et al. Intraoperative methadone for the prevention of postoperative pain: a randomized, double-blinded clinical trial in cardiac surgical patients. Anesthesiology. 2015;122(5):1112–22. doi: 10.1097/ALN.0000000000000633 25837528

[21] Udelsmann A, Maciel FG, Servian DCM, Reis E, de Azevedo TM, Melo M de S. Methadone and morphine during anesthesia induction for cardiac surgery. Repercussion in postoperative analgesia and prevalence of nausea and vomiting. Rev Bras Anestesiol. 2011;61(6):695–701. doi: 10.1016/S0034-7094(11)70078-2 22063370

[22] Wong HMK, Chen PY, Tang GCC, Chiu SLC, Mok LYH, Au SSW, et al. Deep parasternal intercostal plane block for intraoperative pain control in cardiac surgical patients for sternotomy: a prospective randomized controlled trial. J Cardiothorac Vasc Anesth. 2024;38(3):683–90. doi: 10.1053/j.jvca.2023.11.038 38148266

[23] Rowell LN, Mechlin B, Ji E, Addamo M, Girdler SS. Asians differ from non-hispanic whites in experimental pain sensitivity. Eur J Pain. 2011;15(7):764–71. doi: 10.1016/j.ejpain.2010.11.016 21561793 PMC3165029

[24] Watson PJ, Latif RK, Rowbotham DJ. Ethnic differences in thermal pain responses: a comparison of South Asian and White British healthy males. Pain. 2005;118(1–2):194–200. doi: 10.1016/j.pain.2005.08.010 16202529

[25] Chan A, Wong S, Chen PP, Tsoi TH, Lam J, Ip WY, et al. Validation study of the Chinese identification pain questionnaire for neuropathic pain. Hong Kong Med J. 2011;17(4):297–300. 21813898

[26] Yap JC, Lau J, Chen PP, Gin T, Wong T, Chan I, et al. Validation of the Chinese Pain Catastrophizing Scale (HK-PCS) in patients with chronic pain. Pain Med. 2008;9(2):186–95. doi: 10.1111/j.1526-4637.2007.00307.x 18298701

[27] Whitehead AL, Julious SA, Cooper CL, Campbell MJ. Estimating the sample size for a pilot randomised trial to minimise the overall trial sample size for the external pilot and main trial for a continuous outcome variable. Stat Methods Med Res. 2016;25(3):1057–73. doi: 10.1177/0962280215588241 26092476 PMC4876429

[28] Lobova VA, Roll JM, Roll MLC. Intraoperative methadone use in cardiac surgery: a systematic review. Pain Med. 2021;22(12):2827–34. doi: 10.1093/pm/pnab269 34487175

[29] Murphy GS, Szokol JW. Intraoperative methadone in surgical patients: a review of clinical investigations. Anesthesiology. 2019;131(3):678–92. doi: 10.1097/ALN.0000000000002755 31094758

[30] Grant MC, Chappell D, Gan TJ, Manning MW, Miller TE, Brodt JL, et al. Pain management and opioid stewardship in adult cardiac surgery: joint consensus report of the PeriOperative Quality Initiative and the enhanced recovery after surgery cardiac society. J Thorac Cardiovasc Surg. 2023;166(6):1695-1706.e2. doi: 10.1016/j.jtcvs.2023.01.020 36868931

[31] Carvalho AC, Sebold FJG, Calegari PMG, Oliveira de BH, Schuelter-Trevisol F. Comparison of postoperative analgesia with methadone versus morphine in cardiac surgery. Braz J Anesthesiol. 2018;68(2):122–7. doi: 10.1016/j.bjan.2017.09.005 29096877 PMC9391719

[32] Singh K, Tsang S, Zvara J, Roach J, Walters S, McNeil J, et al. Intraoperative methadone use is associated with reduced postoperative pain and more rapid opioid weaning after coronary artery bypass grafting. J Cardiothorac Vasc Anesth. 2024;38(8):1699–706. doi: 10.1053/j.jvca.2024.05.012 38876810

