# Supplementary material for: Intravenous methadone for perioperative acute and chronic pain management in Chinese adult cardiac surgical patients: A protocol for pilot randomized controlled trial
Source: PLoS One. 2025 Jun 2;20(6):e0323820. doi: 10.1371/journal.pone.0323820 (PMC12129159; doi:10.1371/journal.pone.0323820)
Supplement: S1 File — (DOCX) [file pone.0323820.s002.docx]

**Intravenous methadone in perioperative acute and chronic pain management in Chinese adult cardiac surgical patients: a pilot feasibility trial**

1. **BACKGROUND**

**Significance of acute and chronic pain management in cardiac surgery**

Despite modern day improvements in pain treatment and availability of different analgesic modalities, suboptimal postoperative pain control remains an issue in cardiac surgical patients. Acute postoperative pain is common among cardiac surgical patients, particularly within the first 2 days after surgery, with reported at least moderate intensity^1^. There could be many facets for postoperative pain after adult cardiac surgery. Pain can be caused by surgical incisions and dissections, sternal fracture or incomplete bone healing, multiple drainage cannulas and chest tubes and sternal wound infections^2-4^. Poorly controlled acute postoperative pain is associated with adverse physiological outcomes that impair the recovery of cardiac surgical patients. It is associated with decreased patient satisfaction, delayed postoperative ambulation, and the development of chronic postsurgical pain (CPSP)^5^. The association between sternotomy pain and pulmonary complications has been observed, and the sympathetic activation secondary to pain can induce myocardial ischemia and arrhythmias^6-7^. Pain control has also been pointed out as one of the major concerns to cardiac surgical patients in intensive care unit^8^. Therefore, optimal acute pain control not only can improve clinical outcomes, but also improves patient satisfaction after cardiac surgery.

Postoperative pain that persists beyond the normal time for tissue healing is increasingly recognized as an important complication after various types of surgery. According to the International Association for Study of Pain, CPSP is defined as the persistence of pain at surgical site or referred area, at least 3 months following the surgical procedure^9^. CPSP is common after cardiac surgery. The reported incidence was 28% to 56% up to 2 years postoperatively^10-12^. Several mechanisms have been involved in the development of chronic pain after sternotomy. These include dissection, nerve entrapment by sternal wires, sternal retraction, ribs fractures, and intercostal neuralgia as a consequence of nerve damage during dissection of the internal mammary artery during coronary artery bypass graft (CABG)^13-14^. In addition, poorly controlled pain has been a general risk factor for the development of CPSP. All can stimulate the release of pro-inflammatory cytokines which sensitize the afferent nociceptive fibres to cause chronic pain. CPSP has the potential to impact daily functioning and quality of life of patients, as well as increasing the healthcare costs. CARDpain study reported that among those with CPSP, over 50% had significant pain-related interferences with activities of daily living (family and home responsibilities, recreation and employment) at 3, 6 and 12 months following cardiac surgery^15^. Therefore, apart from optimal acute pain control, it is equally important to prevent and manage CPSP, to ensure better satisfaction and quality of lives for our patients.

**Challenges in pain management in cardiac surgical patients**

Intravenous opioids such as fentanyl and morphine have been the mainstay of perioperative analgesia for cardiac surgery, either by intermittent boluses by healthcare staff or through a patient-controlled device (PCA). The primary problem with this mechanism of delivery is that significant fluctuations in serum opioid concentrations can occur, resulting in effects which range from inadequate analgesia to overdose and respiratory depression. These peaks and troughs of analgesia that occur with intermittent opioids administration may explain the suboptimal pain control during the initial postoperative period. In contrast to intermittent administration of short-acting opioids such as morphine and fentanyl, a single dose administration of methadone can be considered. Methadone was conventionally used in cancer and chronic pain management. It can be administered via oral, intravenous, and other parenteral routes. Despite being an often-used alternative to morphine, it remains relatively invisible in perioperative settings. Methadone is a unique opioid that may provide several important potential benefits for surgical patients in the perioperative period. It is a potent mu receptor agonist with a rapid onset and longest half-life (24-36 hours) of the clinically used opioids. According to a pharmacokinetic study^16^, central nervous system effect site methadone concentration rapidly equilibrates with plasma concentrations, evidenced by a short lag time between plasma concentrations and effects (t_1/2_k_e0_ 4min). This is comparable to the rapid onset and effect compartment equilibration of fentanyl and sulfentanil (5-6min), and in contrast the slow onset time of morphine, where t_1/2_k_e0_ has been reported to exceed 4 hours^17^. In addition, as reviewed in an editorial, when methadone is administered at a dose of 20mg or higher, the duration of analgesia approximates the half-life of 24-36 hours. Therefore, a single intravenous dose 20mg administered to an adult at induction of anaesthesia should provide a rapid onset and significant pain relief up to 1-2 days postoperatively, which is the period reported to have the highest pain score after cardiac surgery. Methadone is also a N-methyl-D-aspartate (NMDA) receptor antagonist. It has been reported to possess anti-hyperanalgesic and anti-allodynic properties, that is important in preventing pain sensitization and the development of CPSP^19-20^, which is of high risk in cardiac surgical patients.

**Potential adverse effects of methadone**

Intravenous methadone hydrochloride 10mg/ml will be procured from pharmaceutical company in United States via the Hospital Pharmacy at Prince of Wales Hospital. The clinical trial pharmacy will be involved in dispensing to ensure drug safety during blind labelling. According to the Pain Management Best Practices Inter-Agency Task Force Report^29^ in 2019, methadone is one of the common prescription opioid medications that can be considered for management of acute and chronic pain.

Methadone shares the same opioid-related side effects as with many other opioids such as nausea and vomiting, drowsiness and respiratory depression. According to a meta-analysis^29^ in 2020 which included 7 randomized controlled trials on intraoperative use of intravenous methadone, 4 studies reported no adverse events (respiratory suppression and excessive sedation). One study reported that the patients who received intraoperative methadone experienced more sedation compared to control group at 24 hours after surgery. One study reported that the intraoperative morphine group experienced more sedation compared to methadone group during the postoperative period. Methadone was not shown to have a higher incidence of postoperative nausea and vomiting compared to the morphine group.

Methadone has been reported to be associated with cardiac conduction abnormalities such as QT prolongation, QT interval dispersion and cardiotoxicity (Torsade de pointes)^30^. However, most of the cardiac-related side effects were in patients on prolonged or maintenance treatment with methadone. Significant dose-dependent QTc prolongation usually occurs at a high dose of methadone. A single injection of intravenous methadone at low dose would be unlikely to result in significant cardiotoxicity. No cardiac disturbances were reported from the included studies in the meta-analysis^29^.

All patients will be under full cardiac monitoring (ECG, non-invasive and invasive blood pressure monitoring and transoesophageal echocardiography) throughout the operation. Patient will also be transferred and monitored in intensive care unit for 12 hours after operation and in cardiac high-dependency unit for at least 24 hours after discharge from intensive care.

**Gaps in the literatures**

There have been few randomized controlled trials comparing between intravenous methadone and other opioids for perioperative pain control in cardiac surgery requiring sternotomy, and none in the Asian populations. In addition, the effect of methadone on chronic postsurgical pain in cardiac surgical patients has not been widely reported in literature. Therefore, the primary aim of this pilot randomized controlled trial is a feasibility study to evaluate the protocol and the effect of methadone on acute and chronic pain control after open cardiac surgery, compared with conventional approach of opioid-based analgesia using morphine and fentanyl. In addition, the effects of methadone on opioids consumption, opioid-related side effects, patient satisfaction, postoperative extubation times, and length of stay in hospital and ICU will be determined. We hypothesized that intravenous methadone is associated with a reduction in opioids requirement intraoperatively and in the first 24 hours after surgery, and improvement in acute pain score at 12h after extubation. Our secondary hypothesis is that patients administered with methadone would experience less CPSP compared with standard treatment group.

**Work done by us**

To date, we have worked on individual components in fast-track cardiac anaesthesia to optimize pain management. A pilot study we did has shown that 61% of our cardiac surgical patients suffered from chronic post-surgical pain at 3 months after the operation. The focus of cardiac surgery in Hong Kong has been on the immediate postoperative period and surgical outcomes, rather than on the quality of recovery particularly the pain management as a long-term patient reported outcome measure. A few projects from our research team is going on, targeting at anaesthesia techniques and strategies in optimizing acute and chronic management (eg. regional anaesthesia). Intravenous methadone is a novel approach in Hong Kong.

**Work done by others**

Only a few trials have studied intravenous methadone as an analgesic in surgical patients, in particular, there have not been many studies in the field of cardiac anaesthesia. A randomized controlled trial done by Murphy et al showed intraoperative methadone to be superior to fentanyl for patients undergoing cardiac surgery^23^. Methadone was demonstrated to reduce morphine consumption in the first 24 postoperative hours and improved pain scores 12h after extubation compared to patients receiving fentanyl. There was 40% reduction in morphine requirement during the first 24h after extubation, and the severity of postoperative pain was decreased by 30-40% during the first 3 days after cardiac surgery.

There was only one study the comparing the analgesic efficacy between methadone and morphine given at the time of induction in cardiac surgical patients^24^. Methadone was shown to reduce opioid requirement at 24h postoperatively and significantly reduce the incidence of postoperative nausea and vomiting. So far there has not been any studies on using methadone for cardiac surgery in Chinese populations, and none on the role of methadone for prevention of chronic post-surgical pain in cardiac surgical patients. Substantial literatures have demonstrated the ethnic differences in pain perception and endogenous pain modulation is postulated to be a mechanism for ethnic differences. Studies have compared Caucasians with Asians such as Chinese and Indians. Asians generally demonstrated lower pain tolerance than Caucasians^26-27^.

1. **Aims and hypothesis**

We hypothesized that intravenous methadone is feasible and applicable in cardiac anaesthesia, and is associated with opioid-sparing intraoperatively and within 24h after surgery, as well as better acute and chronic pain control when compared to conventional opioid-based approach with morphine and fentanyl in adult cardiac surgical patients. The aims of the study are as follows:

1. To determine the feasibility of using intravenous methadone in adult cardiac surgical patients
2. To evaluate the effect of methadone in acute and chronic pain management in adult cardiac surgical patients requiring sternotomy
3. **Methods**

*Study design*

This is a single-centre, prospective, randomized-controlled, and double-blinded pilot study. The study was conducted at the Prince of Wales Hospital, a university teaching hospital with 1650 beds in Hong Kong. All elective cardiac surgical patients are admitted to our 23-bed ICU for early postoperative care and monitoring with 1:1 nursing at all times, with an expectation of discharge from ICU to a high-dependency cardiac ward within 24 hours after surgery. Currently, 300-350 adults undergo elective coronary artery bypass graft and/or valvular surgery each year.

This is a pilot feasibility study to determine the effect size of methadone compared with morphine in acute and chronic pain management in cardiac surgery requiring sternotomy. By using a sample size calculator, with an estimated effect size of 0.75 and a significant level of 0.05 using a 2-sided two-sample equal-variance t-test, and to account for a 10% lost to follow-up, a total sample size of 86 (43 per arm) will achieve 90% power to reject the null hypothesis.

*Inclusion and exclusion*

We include adult patients age 18 or older, undergoing elective primary isolated coronary artery bypass grafting, aortic valve repair/replacement, mitral valve repair/replacement or combined coronary artery bypass/valve procedure via sternotomy for the first time, with expected extubation within 12 hours of surgery, in Prince of Wales Hospital, Hong Kong.

The exclusion criteria include: emergency surgery, aortic surgery, preoperative renal failure requiring renal replacement therapy or creatinine clearance <30ml/min (calculated by Cockcroft-Gault Formula), liver dysfunction (liver enzymes twice upper limit normal), LVEF < 40%, requirement of mechanical hemodynamic support in perioperative period, history of chronic pain or who regularly used pain medications (except paracetamol and non-steroidal anti-inflammatory drugs), history of psychiatric illnesses or illicit drug use, intraoperative use of remifentanil, unable to provide informed consent, and history of known or suspected allergy to methadone or other opioid-related drugs.

*Randomization and concealment*

The patients are randomized to receive either methadone 0.2mg/kg or equipotent dose of morphine, added to a syringe containing saline made up to 50ml in total. Randomization will be carried out by an independent investigator, on the day before operation, from pre-prepared envelops each containing the group assignment of either methadone or morphine group. The group assignment will be done by sequence generation with computer models. The study syringes containing the drug solution will be of identical appearance and with blind labelling so that the primary care team and the patient will be blinded to the treatments. An independent assessor blinded to the study will be responsible for the data analysis.

*Blood sampling for drug level*

Venous blood samples (5ml) will be obtained at 0, 30 and 60 minutes after drug administration, and 1.5, 2, 3, 4, 5, 6, 7, 8, 10, 12 hours after drug administration, and 24, 48, 72 and 96 hours after dosing. Plasma concentration of methadone will be determined LC-MS/MS (ESI pos). the internal standard (7-dimethylamino-5,5-diphenyl-4-octanone, 2.5ng) was added to plasma (0.5ml), which was acidified, and then processed by SPE (Waters Oasis MCX cartridges).

*Anaesthesia and interventions*

Intravenous access will be established and all patients will be monitored with standard monitors (non-invasive blood pressure, oxygen saturation, 5-lead ECG, distal radial arterial catheter and Bispectral index). Anaesthesia will be induced with midazolam 0.01-0.05mg/kg, fentanyl 2-5mcg/kg and TCI Propofol 0-1ng/ml Marsh model. Rocuronium 0.5-1mg/kg will be administered to facilitate intubation with single-lumen cuffed endotracheal tube. Anaesthesia is maintained and titrated according to Bispectral index 40-60 with sevoflurane and TCI Propofol infusion. Muscle relaxants are administered to maintain at train-of-four counts less than two. The study drug (either methadone 0.2mg/kg or morphine at equipotent dose) in blind labelling will be administered at time of induction by intravenous infusion over 30 minutes. No further morphine will be given throughout the operation, but administration of intraoperative fentanyl will be left to the discretion of the attending anaesthesiologists. No other analgesics (paracetamol, non-steroidal anti-inflammatory agents, dexmedetomidine, ketamine), steroids or antiemetics will be given intraoperatively. Patient-controlled analgesia (PCA) morphine protocol will be given to patients for 72 hours after operation for postoperative analgesia. Other oral medications for postoperative analgesia will be prescribed by the parent surgical team.

At the end of the operation, the patients will be kept sedated with Propofol infusion to ICU. Propofol infusion is stopped upon admission to ICU to facilitate weaning from ventilator. Adaptive Support Ventilation (ASV) is used in ICU for weaning which adjusts the ventilation parameters depending on the patient’s lung mechanics and effort. Pain is assessed by nurses in ICU after stopping Propofol infusion hourly for 4h, then once every 4h. 1mg morphine will be administered to patients if pain of more than mild severity was noted.

1. **Outcome measures**

*Primary outcomes*

Primary outcomes include the proportion of patients that can be recruited (defined as the number of patients that fits the recruitment criteria divided by the total number of elective cardiac surgical patients over a period of time), proportion of patients excluded based on inclusion and exclusion criteria (with reasons for exclusion), appropriateness of randomization process, the attrition rate (defined as the number of drop out divided by the total number of recruited patients, with reasons for withdrawal recorded), any risks or safety issues arising from this pilot, and the perceived trial feasibility.

*Secondary outcomes*

The secondary outcomes are the time at which the patient is successfully weaned to spontaneous breathing according to ASV; the total morphine consumption within 24h after the operation; intraoperative fentanyl consumption; the total morphine consumption, pain score at rest and upon exertion, and patient satisfaction score to pain within 72h after the operation; time to first morphine rescue; side effects of opioids including the number of episodes of postoperative nausea and vomiting; the recovery from surgery in terms of length of ICU and hospital stay; and psychological distress of pain at 3 and 6 months after surgery. The psychological distress will be assessed using the Neuropathic Pain Scale, Brief Pain Inventory Interference Scale and the Pain Catastrophizing Scale.

1. **Data collection**

All consenting participants will be interviewed on the day before operation. All data is collected by research team members blinded to group assignment. Patient demographics and body mass index will be recorded. The time weaned to spontaneous breathing on ASV will be recorded. At 15min after tracheal extubation and at 12h, 24h, 48h and 72h postoperatively, patients will be asked to quantify the intensity of postoperative pain at rest and upon maximal coughs with numerical rating scale (0 = no pain, 10 = worst pain imaginable). The time to first morphine rescue (in minutes after the operation) will be measured. The level of sedation will be measured using the Ramsay Sedation Scale (1 = anxious, agitated or restless, or both; 2 = co-operative, oriented and tranquil; 3= respond to command only; 4 = exhibit brisk response to light glabellar tap or loud auditory stimulus; 5 = exhibit sluggish response to light glabellar tap or loud auditory stimulus; 6 = exhibits no response), episodes of nausea and vomiting, and whether antiemetics are prescribed will also be recorded at the above time points. At the same time, patients will be asked to rate the overall satisfaction with pain management on a verbal analogue scale (0 = worst possible, 100 = best possible).

The Chinese version of the Neuropathic Pain Questionnaire (NPQ)^28^ and Brief Pain Inventory (BPI) interference scale^21^ will be used to evaluate the presence of neuropathic pain and the extent of pain interfering with various components of functioning, including physical functioning, emotional functioning and sleep within the last 24 hours at 3 and 6 months after surgery. Pain Catastrophizing Scale (HK-PCS)^22^ will be used to assess patients’ negative cognitive-affective response to pain within the last 24 hours at 3 and 6 months after surgery.

The following medical and surgical data during the hospital stays will be extracted from patient charts:

1. Patient demographics (age, gender, EuroScore)
2. Type of surgery
3. Duration of surgery and duration of cardiopulmonary bypass
4. Intraoperative fentanyl (mcg) consumption
5. Daily and total morphine consumption within 72 hours postoperatively
6. ASV time to spontaneous breathing
7. Length of ICU stay
8. Length of hospital stay

|  |  |  | | | | | | | |
| --- | --- | --- | --- | --- | --- | --- | --- | --- | --- |
| Assessment | | Baseline (day 0) | 15min extubation | 12h postop | 24h postop | 48h postop | 72h postop | 3-month postop | 6-month |
| Enrolment | | | | | | | | |  |
| Eligibility screen | | X |  |  |  |  |  |  |  |
| Informed consent | | X |  |  |  |  |  |  |  |
| Demography data | | X |  |  |  |  |  |  |  |
| Comorbidity data | | X |  |  |  |  |  |  |  |
| EuroScore | | X |  |  |  |  |  |  |  |
| Randomization | | X |  |  |  |  |  |  |  |
| Outcomes | | | | | | | | | |
| NRS score | |  | X | X | X | X | X |  |  |
| ASV time to spontaneous breathing | |  | X |  |  |  |  |  |  |
| NPQ | |  |  |  |  |  |  | X | X |
| BPI | |  |  |  |  |  |  | X | X |
| Pain Catastrophizing Scale | |  |  |  |  |  |  | X | X |
| Intraoperative fentanyl consumption | |  | X |  |  |  |  |  |  |
| Time to first morphine rescue | |  |  | X |  |  |  |  |  |
| Postop morphine consumption | |  |  | X | X | X | X |  |  |
| Nausea / vomiting | |  | X | X | X | X | X |  |  |
| Pain satisfaction | |  |  | X | X | X | X |  |  |
| Extubation time | |  | X |  |  |  |  |  |  |
| ICU stay | |  |  |  | X |  |  |  |  |
| Hospital stay | |  |  |  |  |  |  | X |  |

1. **Ethics and statistical analysis**

Patients will be screened for recruitment on the day before operation and explained the risks and benefits of the study. Written informed consent will be obtained from the patient. Data will be kept confidential in securely locked offices of the Department of Anaesthesia and Intensive Care, Prince of Wales Hospital and on secure password-protected computers for 3 years. Access to data will be restricted to study investigators. Identifiable patient data will remain confidential. Ethics approval will be obtained from the Joint CUHK-NTEC Research Ethics Committee. The study will adhere to local laws, Declaration of Helsinki, international Council for Harmonisation of Technical Requirements for Pharmaceuticals for Human Use Good Clinical Practice and institutional policies.

Descriptive and summary statistics will mainly be used for outcome measures. Continuous variables will be expressed as mean ± standard deviation (SD) for normally distributed data or median ± interquartile range (IQR) if not normally distributed. Categorical data will be shown as numbers and percentage. Comparison of continuous data will be performed with Student’s *t* test with normal distribution and by Mann-Whitney U test for non-normally distributed data. Chi-square test will be used to compare groups with categorical variables. P value less than 0.05 is considered significant. Multivariate analysis will be used to find the association between CPSP and methadone use. SPSS 26.0 software (SPSS, Inc, Chicago, IL, USA) is used for data analysis.

1. **References**
2. Mueller XM, Tinguely F, Tevaearai HT, Revelly JP, Chiolero R, von Segesser LK: Pain location, distribution and intensity after cardiac surgery. Chest 2000; 118:391-6
3. Sharma AD, Parmley CL, Sreeram G, Grocott HP. Peripheral nerve injuries during cardiac surgery: risk factors, diagnosis, prognosis, and prevention. Anesth Analg 2000; 91:1358–1369
4. Defalque RJ, Bromley JJ. Poststernotomy neuralgia: a new pain syndrome. Anesth Analg 1989; 69:81–82
5. Kleiman et al. Chroinc Poststernotomy Pain. Regional Anaesthesia and Pain Medicine. Volume 42, Number 6, November-December 2017
6. Kehlet H, Jensen TS, Woolf CJ: Persistent postsurgical pain: Risk factors and prevention. Lancet 2006; 367:1618-25
7. Puntillo K, Weiss SJ: Pain: its mediators and associated morbidity in critically ill cardiovascular surgical patients. Nurs Res 1994; 43:31-6
8. Roediger L, Larbuisson R, Lamy M: New approaches and old controversies to postoperative pain control following cardiac surgery. Eur J Anaesthesiol 2006; 23:539-50
9. Chyun D. Patient perception of stressors in intensive care and coronary care units. Focus Crit Care 1989; 16:206-211
10. Werner MU, Kongsgaard UE. I. Defining persistent postsurgical pain: is an update required? Br J Anaesth. 2014;113:1–4
11. Eisenberg E, Pultorak Y, Pud D, Bar-El Y. Prevalence and characteristics of post coronary artery bypass graft surgery pain (PCP). Pain 2001; 92:11– 17
12. Kalso E, Mennander S, Tasmuth T, Nilsson E. Chronic poststernotomy pain. Acta Anaesthesiol Scand 2001; 45:935–939
13. Meyerson J, Thelin S, Gordh T, Karlsten R. The incidence of chronic poststernotomy pain after cardiac surgery-a prospective study. Acta Anaesthesiol Scand 2001; 45:940–944.
14. Sharma AD, Parmley CL, Sreeram G, Grocott HP. Peripheral nerve injuries during cardiac surgery: risk factors, diagnosis, prognosis, and prevention. Anesth Analg 2000; 91:1358–1369
15. Defalque RJ, Bromley JJ. Poststernotomy neuralgia: a new pain syndrome. Anesth Analg 1989; 69:81–82
16. Choinière M, Watt-Watson J, Victor JC, *et al*. Prevalence of and risk factors for persistent postoperative nonanginal pain after cardiac surgery: a 2-year prospective multicentre study. *CMAJ* 2014;186:E2 13– E223.
17. Inturrisi CE, Colburn WA, Kaiko RF, Houde RW, Foley KM. Pharmacokinetics and pharmacodynamics of methadone in patients with chronic pain. Clin Pharmacol Ther. 1987; 41:392-401
18. Dershwitz M, Walsh JL, Morishige RJ, Connors PM, Rubsamen RM, Shafer SL, Rosow CE. Pharmacokinetics and pharmacodynamics of inhaled versus intravenous morphine in healthy volunteers. Anesthesiology. 2000; 93:619-28
19. Kharasch ED. Intraoperative methadone: Rediscovery, reappraisal, and reinvigoration? Anesth Analg 2011; 112:13-6
20. Gagnon B, Almahrezi A, Schreier G. Methadone in the treatment of neuropathic pain. Pain Res Manag 2003; 8:149-54
21. Santiago-Palma J, Khojaninova N, Kornick C, et al. Intravenous methadone in the management of chronic cancer apin: safe and effective starting doses when substituting methadone for fentanyl. Cancer 2001; 92:1919-25
22. Ger LP, Ho ST, Sun WZ, Wang MS, Cleeland CS. Validation of the brief pain inventory in a Taiwanese population. J Pain Symptom Manage. 1999 Nov;18(5):316-22
23. Validation of the Chinese Pain Catastrophizing Scale (HK-PCS) in Patients with Chronic Pain. Pain Medicine. Vol 9, Number 2, 2008
24. GS Murphy, JW Szokol, MJ Avram, JH Marymont, T Shear, KN Parikh, SS Patel, DK Gupta. Intraoperative methadone for the prevention of postoperative pain. Anaesthesiology. Vol 122, Number 5, 2015
25. A Undelsmann et al. Methadone and morphine during anaesthesia induction for cardiac surgery. Repercussion in postoperative analgesia and prevalence of nausea and vomiting. Rev Bras Anestesiol 2011; 61:6:695-701
26. Yau DKW, Wong MKH, Wong WT, Gin T, Underwood MJ, Joynt GM, Lee A. PREhabilitation for improving QUality of recovery after ELective cardiac surgery (PREQUEL) study: protocol of a randomised controlled trial. BMJ Open. 2019 May 14;9(5):e027974. doi: 10.1136/bmjopen-2018-027974.
27. Rowell LN, Mechlin B, Ji E, et al. Asians differ from non-Hispanic Whites in experimental pain sensitivity. Eur J Pain. 2011;15(7):764–771. doi:10.1016/j.ejpain.2010.11.016
28. Watson PJ, Latif KR, Rowbotham DJ. Ethnic differences in thermal pain responses: a comparison of South Asian and White British healthy males. Pain. 2005;120:194–220. doi:10.1016/j.pain.2005.08.010
29. SJ Krause, MM Backonja. Development of a neuropathic pain questionnaire. The Clinical Journal of Pain 2003; 19:306-314
30. Pain Management Best Practices Inter-Agency Task Force. US Department of Health and Human Services (2019, May). Pain Management Best Practices Inter-Agency Task Force Report: Updates, Gaps, Inconsistencies, and Recommendations. Retrieved from US Department of Health and Human Services website: <https://www>.hhs.gov.ash/advisory-committees/pain/reports/index.html
