# Supplementary material for: Intravenous methadone for perioperative acute and chronic pain management in Chinese adult cardiac surgical patients: A protocol for pilot randomized controlled trial
Source: PLoS One. 2025 Jun 2;20(6):e0323820. doi: 10.1371/journal.pone.0323820 (PMC12129159; doi:10.1371/journal.pone.0323820)
Supplement: S2 File — (PDF) [file pone.0323820.s003.pdf]

香港中文大學醫學院
Faculty Of Medicine
The Chinese University Of Hong Kong

醫院管理局
新界東醫院聯網
Hospital Authority
New Territories East Cluster

Joint Chinese University of Hong Kong-New Territories East Cluster Clinical Research Ethics Committee
香港中文大學-新界東醫院聯網 臨床研究倫理 聯席委員會

8/F, Lui Che Woo Clinical Sciences Building, Prince of Wales Hospital, Shatin, HK
Tel : (852) 3505 3935 / 2144 5926 Fax : (852) 2646 6653 Website : http://www.crec.cuhk.edu.hk

The Joint CUHK-NTEC CREC is an independent committee established by CUHK/NTEC and authorized to perform ethics and scientific review and oversight of clinical studies within the jurisdiction of CUHK/NTEC in accordance with its standard operating procedure and the principles of the Declaration of Helsinki and ICH Good Clinical Practice.

CREC Ref. No.: 2022.636-T

18 JAN '23

To: Dr. Man Kin WONG
Dept. of Anaesthesia & Intensive Care
Prince of Wales Hospital

This notice is issued by the Joint CUHK-NTEC CREC with respect to the application/submission by you, being the principal investigator of the following study at your study site:

- **Study Protocol Title:** Intravenous methadone in perioperative acute and chronic pain management in Chinese adult cardiac surgical patients: a pilot feasibility trial
- **Investigator(s):** Man Kin WONG, XiaoDong LIU, Ara Cheuk Yin LI, Sylvia Siu Wah AU, Randolph Hung Leung WONG and Wai Tat WONG

In accordance with our standard operating procedure, we have duly performed ethics and scientific review of your application/submission as detailed below:

- **Nature of Your Application/Submission:** ☒ Initial application ☐ Amendments/changes ☐ Others: ☐ Renewal
- **Mode of Review:** ☒ Full review ☐ Expedited review
- **Date of Initial/Renewal Approval:** 12 January 2023
- **Document(s) Reviewed:** See Schedule 1
- **Reviewer(s):** See Schedule 2

After due review by our reviewer(s), we hereby write to inform you of our decision on your application/submission as follows:

- **Decision:**
  - ☐ Application/Submission approved
  - ☒ Application/Submission approved with condition(s) (see condition(s) below)
  - ☐ Application/Submission approved with remark(s) (see remark(s) below)
  - ☐ Application/Submission approved with condition(s) and remark(s) (see condition(s) and remark(s) below)
- **Condition(s):** A copy of the Certificate for Clinical Trial is required to be submitted to the Joint CUHK-NTEC CREC prior to commencement of the study

18 JAN '23

- **Regular Progress Report(s) Required:** Every 12 months from the date of initial/renewal approval and during the period of the study if required

You, being the principal investigator of the study at your study site, are reminded to comply with our requirements and to maintain communication with us during the period of the study by undertaking the principal investigator's responsibilities including (but not limited to):

- if the study is an industry-sponsored clinical study, submitting to us a copy of the fully executed indemnity agreement satisfying the Hospital Authority's requirement prior to commencement of the study (if it has not been submitted yet);
- observing and complying with all applicable requirements under our standard operating procedure ("IRB/REC SOP"), the Declaration of Helsinki and the ICH GCP (if applicable);
- submitting regular progress report(s) at the required intervals (as specified above) in accordance with the requirements in the IRB/REC SOP;
- not implementing any amendment/change to any approved study document/material without our written approval, except where necessary to eliminate any immediate hazard to the subjects or if an amendment/change is only of an administrative or logistical nature;
- notifying us of any new information that may adversely affect the rights, safety or well-being of the subjects or the proper conduct of the study;
- reporting any deviation from the study protocol or compliance incident that has occurred during the study and may adversely affect the rights, safety or well-being of any subject in accordance with the requirements in the IRB/REC SOP;
- submitting safety reports on all SAEs observed at your study site or SUSARs reported from outside your study site in accordance with the requirements in the IRB/REC SOP; and
- submitting a final report in accordance with the requirements in the IRB/REC SOP upon completion or termination of the study at your study site.

In addition to the above, you are also reminded to observe and comply with other applicable regulatory and management requirements including (but not limited to):

- if required by Hong Kong laws or regulations, obtaining a certificate for clinical trial through the Hong Kong Department of Health and complying with the associated requirements;
- obtaining the necessary consent from the management of your institution/department in accordance with the requirements of your institution/department;
- if required by local laws or regulations at conducting site out of IRB/REC's jurisdiction, obtaining an approval and complying with associated requirements;
- not representing to any third party or in any way likely to mislead any third party forming the view that the approval from the IRB/REC has any extraterritorial effect; and
- with due diligence ensuring your teams, staff, agents or whosoever connected with you to comply with the preceding requirements.

Yours sincerely,

Envy Lee (Secretary)
for and on behalf of
The Joint CUHK-NTEC CREC

EL/ci

Schedule 1
Documents Reviewed

The documents reviewed by with respect to the said application/submission include:

- Protocol, Version 2.0, dated 06 January 2023
- Patient Information Sheet and Consent Form, English and Chinese Version 3.0, dated 12 January 2023
- Neuropathic Pain Questionnaire, English Version 1.0, dated 06 January 2023
- Neuropathic Pain Questionnaire, Chinese Version 1.0, dated 06 January 2023
- Pain Catastrophizing Scale, Chinese Version 1.0, dated 06 January 2023
- Brief Pain Inventory, Chinese Version 1.0, dated 06 January 2023

Schedule 2
Reviewers List – Group 1
Joint CUHK-NTEC Clinical Research Ethics Committee

Title and Name	Occupation	Qualification	Male / Female (M/F)	Study Reviewed by	Present in CREC meeting on 03 Jan 2023
**Chairman:** Prof. Cheuk Chun SZETO	Professor, Department of Medicine and Therapeutics, PWH, CUHK	MBChB(Hons)(CUHK), MD(CUHK), FRCP(Edin & Lond), FHKCP, FHKAM	M	√	√
Prof. Wai Kwong TANG	Professor, Department of Psychiatry, CUHK	MBChB(CUHK), MD (CUHK), MRCP(UK), FHKCP, FHKAM	M	√	
Prof. Tsz Ping LAM	Associate Professor (Clinical) of Orthopaedics and Traumatology, CUHK	MBBS(HKU), FRCS(Edin), FHKCS, FHKCOS, FHKAM(Ortho Surg)	M	√	√
Prof. Martin C.S. WONG	Associate Director (General Affairs) JC School of Public Health and Primary Care, CUHK	MD, MBChB (CUHK), BMedSc (Hons), MSc(Hons), MPH(CUHK), MBA, FHKCFP, FRACGP(Aust), FRSPH(UK), FFPH, FESC, FACC, FAcadTM, DCH (Ireland), FHKAM(Fam Med), HKAN (Hon Fellow), FRCP (Glasgow, Edin)	M	√	
Prof. Benny C.Y. ZEE	Professor, School of Public Health, CUHK	BSc(Manitoba), MSc(Manitoba), PhD(Pittsburgh)	M		
Dr. Shannon Melissa CHAN	Assistant Professor, Department of Surgery, CUHK	MBChB, MRCS (Edin), FRCS (Edin), FHKAM (Surgery), FCSHK	F	√	
Dr. Assunta Chi Hang HO	Associate Consultant, Department of Paediatrics, PWH	MBChB, MRCPCH, DCH, FHKCP, FHKAM(Paed)	F		√
Dr. Billy Ming Hei LAI	Consultant<br>Deputy Coordinator for Angiography Services<br>Clinical Associate Professor (Honorary)<br>Department of Imaging and Interventional Radiology<br>Prince of Wales Hospital, The Chinese University of Hong Kong	MBBS, MRes(Med), FRCR, FHKCR, FHKAM(Radiology)	M		
Dr. Agnes S.Y. LEUNG	Clinical Lecturer Department of Paediatrics CUHK	MB ChB (CUHK), MRCPCH (UK), FHKCPaed, FHKAM (Paediatrics)	F	√	√
Dr. Eddy H. K. SIU	Manager, Physiotherapy Department, PWH	PhD (Physiotherapy), MSc (Manip Physiotherapy), MSc (Ex & Nutr Sci), BSc (Physiotherapy)	M		

18 JAN '23

Title and Name	Occupation	Qualification	Male / Female (M/F)	Study Reviewed by	Present in CREC meeting on 03 Jan 2023
Ms. Man Ching LAW	Nurse from Dept. of Medicine and Therapeutics, PWH	RN, BN, MSocSc(Couns), ADipHSM, Cert Renal Nsg, MHKPCA, FHKAN (Medicine - Renal), FHKAN (Education & Research - Education)	F	√	√
Dr. Alfred H.M CHAN	Senior Administration Officer, Academy of International Dispute Resolution and Professional Negotiation	PhD(CUHK), IME, MHKMC, CWPM, IPDRA	M	√	√
Dr. Grace S.N. LAU	Part Time Lecturer	BPharm(Hons), PhD	F	√	√
Ms. Katrina P.Y. KWAN	Solicitor	Postgraduate Certificate in Laws – PCLL<br>Bachelor of Laws (Honours) – LL.B. (Hons.)	F	√	
Mr. Jacob C.H. LEE	Senior Vice President, Chow Tai Fook Enterprises Limited	BBA (Univ. of Michigan), Master of Science in Accounting and Finance (LSE, Univ. of London), CFA	M	√	√
Ms. Kitty Y.C. NG	Associate (Corporate / Cross Border Transactions)	LLB (CUHK), PCLL (CUHK), LLM (Cantab)	F		
Ms. Asha SHARMA	Partner, ReedSmith Richards Butler LLP	Solicitor (HK and England & Wales), CIArb member, Mediator (Law Society HK and HKMAAL), PCLL (HKU), LLB (University of London), LE (PolyU)	F	√	√
Ms. May Ling WONG	Partner, ReedSmith (Driving progress through partnership)	BSc, LLB, PCLL (HKU)	F	√	√
