# Supplementary material for: Intravenous methadone for perioperative acute and chronic pain management in Chinese adult cardiac surgical patients: A protocol for pilot randomized controlled trial
Source: PLoS One. 2025 Jun 2;20(6):e0323820. doi: 10.1371/journal.pone.0323820 (PMC12129159; doi:10.1371/journal.pone.0323820)
Supplement: Peer review history

# Author comment: Author response to Decision Letter 0

**DOI:** 10.1371/journal.pone.0323820.r001

5 Apr 2025

# Editor report: Decision Letter 0

**DOI:** 10.1371/journal.pone.0323820.r002

**Authors:** Steve Zimmerman

16 Apr 2025

Intravenous methadone for perioperative acute and chronic pain management in Chinese adult cardiac surgical patients: a protocol for pilot randomized controlled trial

PONE-D-25-16024

Dear Dr. WONG,

As a Peer-Reviewed Funded Protocol, your submission is eligible for expedited review by in-house editors. Based on our evaluation, we are satisfied that your manuscript meets our publication criteria for Study Protocols, and is therefore considered to be suitable for publication subject to final journal requirements.

Within one week, you’ll receive an e-mail detailing the required amendments. When these have been addressed, you’ll receive a formal acceptance letter and your manuscript will be scheduled for publication.

An invoice will be generated when your article is formally accepted. Please note, if your institution has a publishing partnership with PLOS and your article meets the relevant criteria, all or part of your publication costs will be covered. Please make sure your user information is up-to-date by logging into Editorial Manager at Editorial Manager®  and clicking the ‘Update My Information' link at the top of the page. If you have any questions relating to publication charges, please contact our Author Billing department directly at authorbilling@plos.org.

If your institution or institutions have a press office, please notify them about your upcoming paper to help maximize its impact. If they’ll be preparing press materials, please inform our press team as soon as possible -- no later than 48 hours after receiving the formal acceptance. Your manuscript will remain under strict press embargo until 2 pm Eastern Time on the date of publication. For more information, please contact onepress@plos.org.

Kind regards,

Steve Zimmerman, PhD

Senior Editor, PLOS One

# Editor report: Acceptance letter

**DOI:** 10.1371/journal.pone.0323820.r003

**Authors:** Steve Zimmerman

PONE-D-25-16024

PLOS ONE

Dear Dr. WONG,

I'm pleased to inform you that your manuscript has been deemed suitable for publication in PLOS ONE. Congratulations! Your manuscript is now being handed over to our production team.

At this stage, our production department will prepare your paper for publication. This includes ensuring the following:

* All references, tables, and figures are properly cited

* All relevant supporting information is included in the manuscript submission,

* There are no issues that prevent the paper from being properly typeset

You will receive further instructions from the production team, including instructions on how to review your proof when it is ready. Please keep in mind that we are working through a large volume of accepted articles, so please give us a few days to review your paper and let you know the next and final steps.

Lastly, if your institution or institutions have a press office, please let them know about your upcoming paper now to help maximize its impact. If they'll be preparing press materials, please inform our press team within the next 48 hours. Your manuscript will remain under strict press embargo until 2 pm Eastern Time on the date of publication. For more information, please contact onepress@plos.org.

If we can help with anything else, please email us at customercare@plos.org.

Thank you for submitting your work to PLOS ONE and supporting open access.

Kind regards,

PLOS ONE Editorial Office Staff

on behalf of

Dr Steve Zimmerman

Staff Editor

PLOS ONE
